# Exosomal DNAJB11 promotes the development of pancreatic cancer by modulating the EGFR/MAPK pathway

**DOI:** 10.1186/s11658-022-00390-0

**Published:** 2022-10-08

**Authors:** Peng Liu, Fuqiang Zu, Hui Chen, Xiaoli Yin, Xiaodong Tan

**Affiliations:** 1 Department of General Surgery, Shengjing Hospital of China Medical University, Shenyang, 110004 China; 2 Diagnostic and Therapeutic Center of Pancreatic Diseases of Liaoning Province, Shenyang, 110004 China; 3 Department of Radiology, Shengjing Hospital of China Medical University, Shenyang, 110004 China

**Keywords:** Pancreatic cancer, Exosomal protein, Signal transduction, Early diagnosis

## Abstract

Pancreatic ductal adenocarcinoma (PDAC) is a malignant tumor with invasive and metastatic characteristics and poor prognosis. Intracellular protein homeostasis is associated with invasion and metastasis of pancreatic cancer, but the specific molecular mechanism remains unclear. Our previous studies have revealed that DNAJB11, a key protein in protein homeostasis, is secreted by exosomes in the supernatant of dissociated pancreatic cancer cells with high metastasis. The results from transcriptome sequencing and co-immunoprecipitation (Co-IP)-based liquid chromatography with tandem mass spectrometry (LC–MS/MS) showed that depletion of DNAJB11 levels could increase HSPA5 expression and induce endoplasmic reticulum stress through the PRKR-like endoplasmic reticulum kinase signaling pathway in pancreatic cancer cells. Furthermore, exosomal DNAJB11 promoted cell development of PC cells in vitro and in vivo. In addition, exosomal DNAJB11 could regulate the expression of EGFR and activate the downstream MAPK signaling pathway. Clinical blood samples were collected to evaluate the potential of exosome DNAJB11 as a diagnostic biomarker and therapeutic target for the treatment of pancreatic cancer. This study could provide a new theoretical basis and potential molecular targets for the treatment of pancreatic cancer.

**Supplementary Information:**

The online version contains supplementary material available at 10.1186/s11658-022-00390-0.

## Background

Pancreatic cancer (PC) is a lethal malignancy with low resection and high recurrence rates. In addition, PC is dormant at the early stage, with an overall 5-year survival rate of < 5% [1]. However, since 2000, the PC incidence has increased by approximately 1% per year. Furthermore, by 2030, PC is expected to be the second leading cause of cancer deaths worldwide [2, 3]. Current treatment options, including surgical resection, conventional chemotherapy, radiotherapy, and combination therapies, remain suboptimal [4], probably owing to the tumor microenvironment (TME) of PC [5]. Therefore, it is important to discuss the cancer progression mechanism and identify novel biomarkers for the early prevention and detection of PC.

Exosomes are cup-like extracellular vesicles 40–150 nm in diameter that transmit functional cargoes (proteins, mRNAs, and miRNAs) to recipient cells and regularize their functioning or pathological progression [6]. Accumulating evidence supports that exosomes could affect the TME and play an important role in tumor progression and metastasis [7, 8]. Furthermore, the expression level of pancreatic cancer-derived exosomes is different from patients without PC [9]. The endoplasmic reticulum (ER) is an important organelle in cells that coordinates protein folding and is the point of origin for protein secretion. The ER, like a senior engineer, maintains protein homeostasis through quality control [10]. The unfolded protein response (UPR) is activated when the dynamic process is broken (ER stress). Cancer cells are exposed to multiply factors and trigger the UPR. Then, the UPR stimulates the transcription of genes required for tumor survival and growth [11, 12]. Heat Shock Protein Family A (Hsp70) Member 5 (HSPA5, also called BiP or GRP78) is a major regulator of ER homeostasis and controls the activation of transmembrane endoplasmic reticulum stress (ERS) sensors (e.g., IRE1, ATF6, and PERK) [13]. HSPA5 is responsible for maintaining the “stemness” properties in PC attributed to destructive properties like metastasis [14]. In our previous study, the DnaJ Heat Shock Protein Family (Hsp40) Member B11 (DNAJB11), a co-chaperone for HSPA5, recruited HSPA5 and other chaperones to the substrate for ERAD (ER-associated degradation) to activate UPR [15], was secreted to the extracellular region through exosome method. There was a significant difference in expression levels between the conditioned medium (CM) of the invasive and the less invasive pancreatic cancer cell lines [16]. Hence, DNAJB11 may contribute to the progression and metastasis of PC. However, the molecular mechanisms underlying the efficacy of exosomal DNAJB11 in PC have not been characterized.

In this study, we revealed that exosomal DNAJB11 can enhance the invasive ability of poorly invasive PC cells, and further explained the effect of UPR in pancreatic cancer. Next, exosomal DNAJB11 contributed to cancer progression, and exosomal DNAJB11 can act as a diagnostic biomarker for PC.

## Materials and methods

### Ethics statement and human plasma

Ethics approval was obtained from the committee of the Ethical Committee of the Affiliated Shengjing Hospital of China Medical University with certificate number 2019PS481K for the animal studies and 2017PS46K for the blood specimens. In total, 90 plasma specimens were collected in the study. Before collecting blood specimens, no patients received other treatment like chemotherapy, radiotherapy, or surgery. All the patients and healthy individuals provided informed written consent. All animal experiments were conducted following the standards of the Ethical Committee of Shengjing Hospital.

### Cell lines and cell culture

The culture conditions of the cells were as previously described [17]. AsPC-1 (cat. no. TCHu 8), Capan-2 (cat. no. SCSP-568), BxPC-3 (cat. no. TCHu 12), and MIA Paca-2 (cat. no. SCSP-568) were purchased from the Institute of Biochemistry and Cell Biology, Chinese Academy of Sciences (Shanghai, China). HPDE6-C7 (H6c7, cat. no. ECA001), which is a normal pancreatic duct epithelial cell line, was purchased from Kerafast (Boston, MA, USA). The cells were cultured in RPMI-1640 (Gibco-BRL, Grand Island, NY, USA) supplemented with 10% fetal bovine serum + 1% streptomycin. Cells were maintained at 37 °C in an atmosphere containing 5% CO_2_.

### Exosomes extraction from plasma and medium, TEM, and NTA analysis

Plasma exosomes were isolated from PC, benign pancreatic disease, and healthy individuals by ExoQuick (SBI, CA, USA) following the manufacturer’s instructions [18]. Cell culture medium from each cell line was used for exosome isolation using the differential ultracentrifugation method. The exosome pellets were resuspended in 1 ml of PBS. Purified exosomes (10 μg) from each cell resuspended in 500 μl PBS (Gibco-BRL, NY, USA) were used to treat correspondence cells according to the assay. Exosomes were prepared for identification by western blotting, transmission electron microscopy (TEM), and nanoparticle tracking analysis (NTA). Exosomes were visualized using TEM (JEM1230, JEOL, Akishima, Japan). The concentration of the samples (particles/mL) and size distribution (in nanometers) of the exosome samples were determined using ZetaView (Particle Metrix, Meerbusch, Germany) and the corresponding ZetaView 8.04.02. software.

### Tissue microarray

The tissue microarray (TMA) containing 90 PC tissue samples and 56 matched adjacent paracancerous tissue samples with survival times was purchased from Outdo Biotech (Shanghai, China). The samples were collected from November 2004 to December 2008. The immunohistochemistry assay and the analysis of clinicopathological features were based on tissue microarray. Detailed clinical and pathologic information of patients is presented in Additional file 2: Table S2. The intensity of staining was divided into four scores: 0 (none), 1 (weak), 2 (moderate), and 3 (strong). The immunohistochemistry results were expressed as the percentage of positive cells and staining intensity. The cutoff score was 2.0, and a score of ≥ 2.0 was defined as positive.

### Construction of DNAJB11-knockdown cells in AsPC-1 cells

Lentiviruses containing shRNA targeting human DNAJB11 (Hanheng Biotechnology, Shanghai, China) were transfected into AsPC-1 to knock down DNAJB11. Stable transfection with a lentiviral vector was performed following the manufacturer’s protocols. Knockdown efficacy was determined by quantitative polymerase chain reaction (qPCR) and western blot.

### Transcriptome sequencing

Sequencing was performed by Amogene (Xiamen, China). First, total RNA was quantified and purified. Three micrograms of RNA of each sample was used for RNA-seq library preparation. Second, RNA-seq libraries were conducted using the NEBNext Ultra II RNA Library Prep Kit (NEB, E7760, Illumina, San Diego, CA, USA) according to the manufacturer’s protocol. In brief, poly-T oligo-attached magnetic beads were used to purify mRNA. First- and second-strand cDNA synthesis was performed. The library fragments were analyzed by AMPure XP system (Beckman Coulter, Brea, CA, USA). Next, PCR was conducted with universal PCR primers, index (X) primer, and Phusion High-Fidelity DNA polymerase. Finally, PCR products were purified, and RNA integrity was assessed on an Agilent 2100 Bioanalyser (Agilent, Santa Clara, CA, USA).

For the quantification of gene expression level and differential expression analysis, the read numbers mapped to each gene were counted using Cufflinks V2.2.1.(http://cole-trapnell-lab.github.io/cufflinks/) [19]. Next, the fragments per kilobase of transcript per million mapped reads of each gene was calculated on the basis of gene length and its mapped read counts. Finally, differentially expressed genes (DEGs) were analyzed using Cufflinks. The Benjamini–Hochberg correction was performed to adjust the *P*-values. Corrected *P*-value < 0.05 and absolute fold change > 1.5 were selected as the threshold of differential expression.

### Co-IP-based LC–MS/MS

Co-immunoprecipitation (Co-IP) was performed following the instructions on the cross-linking CoIP kit (Thermo Fisher, Waltham, MA, USA). First, the cells were lysed with 500 µL RIPA buffer containing protease and phosphatase inhibitor on ice for 30 min. Next, the cell lysate supernatant was collected by centrifugation and incubated with anti-DNAJB11 antibody overnight at 4 °C. Next, protein A agarose was added, and cell lysates were further mixed and incubated for 4 h, after being centrifuged at 3,000*g* for 3 min and washed thrice with lysis buffer. Finally, the bound proteins were eluted by boiling for 5 min in 2× loading buffer and separated by SDS-PAGE followed by liquid chromatography and tandem mass spectrometry (LC–MS/MS). LC–MS/MS was performed as previously described with minor modifications [16]. Protein digestion was performed following the standard protocol, and 5 μL of each sample was loaded for LC–MS/MS. The Eksigent nanoLC 415 high-performance liquid chromatograph (Eksigent, Redwood, CA, USA) with the 5600 + TripleTOF (AB Sciex, Framingham, CA, USA) was used to analyze protein fractions. Mobile phases consisted of A (98% H_2_O and 2% acetonitrile with 0.1% formic acid) and B (2% H_2_O and 98% acetonitrile with 0.1% formic acid). Peptides were loaded onto a C18 trap column (100 μm i.d. ×  2 cm) and separated by a capillary separation column (75 μm i.d. ×  15 cm) at 0.3 μL/min with a 90 min gradient. The MS data were acquired using Data Independent Acquisition (DIA) mode, 30 data-dependent MS/MS scans per full scan, rolling collision energy, dynamic exclusion (exclusion duration 15 s), MS/MS scan range of 100–1500 *m*/*z*, and scan time of 50 ms. The ProteinPilot software (Version 4.5, ABSciex) was used for data analysis. The Unused Protein Score was used to measure protein confidence.

### Measurement of ECAR

Glycolytic ability was measured using a Seahorse XF96 analyzer (Seahorse Biosciences, Agilent) according to the manufacturer’s instructions. Twenty-thousand cells per well were plated in 96-well XF microplates and cultured for 6 h. All measurements were recorded at set time intervals and normalized to total protein content. Extracellular acidification rate (ECAR) after oligomycin treatment indicates glycolytic capacity. ECAR was calculated using the Wave software.

### Western blot, immunohistochemical assay

Western blotting was performed as described previously [20]. Samples of equivalent total protein (20 μg) were loaded. Primary antibodies against CD63, CD9 (Santa Cruz Biotechnology, Santa Cruz, CA, USA), c-MYC, p-EGFR, p-MEK, p-ERK(Cell Signaling Technology, Danvers, MA, USA), GAPDH, DNAJB11, HSPA5, ATF6, IRE1, XBP1, PERK, ATF4, EGFR, Raf-1, MEK, and ERK1/2 (ProteinTech Group, Rosemont, IL, USA) were used. Immunohistochemical (IHC) assay was performed following a previously described procedure [21].

### Colony formation, invasion and migration assays, cell apoptosis

A colony formation assay was performed to assess cell proliferation. Transwell and wound healing assays evaluated cell invasion and migration. The assays were performed as previously described [21]. Apoptosis assay was performed using an apoptosis detection kit according to the manufacturer’s protocol. The cells were cultured in six-well plates at a density of 1 × 10^5^ cells per well. Following digestion with trypsin and washing twice with PBS, the cells pellet were incubated with 5 µl Annexin V-FITC and 5 µl PI for 15 min at room temperature in the dark. The cell suspensions were analyzed by flow cytometry (FACScan; BD Biosciences) and FlowJo software (version 10.0; FlowJo LLC). The total amount of apoptosis was the results of early apoptosis plus late apoptosis.

### Animal study

The subcutaneous tumor model was used to evaluate cell proliferation in vivo according to a previously described method [20]. First, 18 4-week-old female BALB/c nude mice were obtained from Huafukang Biotechnology Co (Beijing, China). Next, 1 × 10^6^ cells from each group in 0.1 mL PBS were injected into the right axillary region of each mouse to generate 60 mm^3^ tumors. The tumor diameter was measured twice a week. Volume was calculated as volume = 0.5 × *L* × *W*^2^, where *L* is the long axis of the tumor and *W* is the short axis of the tumor.

### Enzyme-linked immunosorbent assay (ELISA)

The DNAJB11 protein level of plasma-derived exosomes was determined using the human DNAJB11 ELISA kit (Cloud-Clone, Wuhan, China) following the manufacturer’s protocol.

### Bioinformatic analyses

The Gene Expression Profiling Interactive Analysis (GEPIA) database (http://gepia.cancer-pku.cn) provided the DEGs based on the integrated RNA-seq dataset from Genotype-Tissue Expression (GTEx) databases and The Cancer Genome Atlas (TCGA) [22]. UALCAN (http://ualcan.path.uab.edu/index.html) [23] was used to assist DNAJB11 expression across TCGA cancers. The Kaplan–Meier (KM) plotter database included the genes that were associated with overall survival (OS) and relapse-free survival (RFS) of patients’ information [24]. The R packages “clusterProfiler” and “ggplot2” were used to run and visualize Gene Ontology (GO) analysis, KEGG pathways, and Gene Set Enrichment Analysis (GSEA). GO terms with corrected *P*-value less than 0.05 were significantly enriched by differentially expressed genes. Multivariate Cox analysis was performed to identify the independent prognostic factor on the basis of the common clinicopathological data.

### Statistical analysis

The R version 3.6.1 and RStudio software were used for statistical analyses and figure outputs. Other statistical analyses and graphics were performed and generated using GraphPad Prism 9.0. The data were analyzed using two-tailed Student’s *t*-test and one-way analysis of variance (ANOVA). *P*-value < 0.05 was considered statistically significant in this study.

## Results

### Exosomal DNAJB11 from plasma obtained from patients with PC was correlated with pancreatic cancer progression

**Fig. 1:**
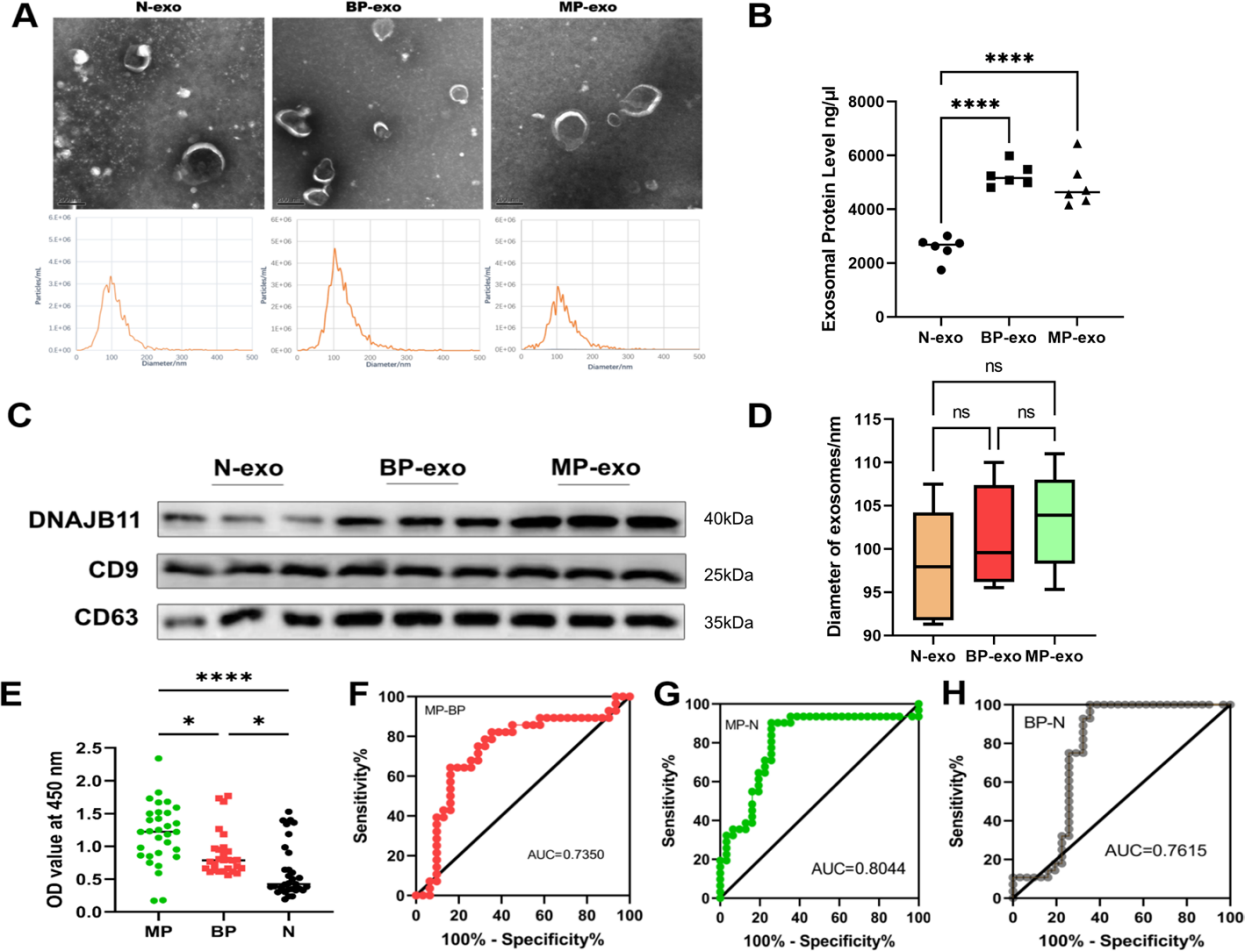
Exosomal DNAJB11 is a novel diagnostic biomarker for pancreatic cancer. **A** TEM and NTA of exosomes from healthy people (N-exo), patients with benign pancreatic disease (BP-exo), and patients with malignant pancreatic cancer (MP-exo) are shown. **B** Protein levels of N-exo, BP-exo, and MP-exo. (*n* = 6 per group). **C** Western blot validation of exosomal markers (CD9 and CD63) and DNAJB11 of N-exo, BP-exo, and PC-exo (*n* = 6 per group). **D** No significant difference between the sizes of N-exo, BP-exo, and PC-exo (*n* = 6 per group). **E** Exosomal DNAJB11 level distribution in clinical plasma samples. **F** ROC curve analysis of exosomal DNAJB11 between MP-exo and BP-exo groups. **G** ROC curve analysis of exosomal DNAJB11 between MP-exo and N-exo groups. **H** ROC curve analysis of exosomal DNAJB11 between BP-exo and N-exo groups. *P < 0.05, **P < 0.01, ***P < 0.001, ****P < 0.0001, one-way ANOVA. The values are shown as mean ± standard error of the mean (SEM)

Plasma exosomes from patients with malignant pancreatic cancer (MP-exo), patients with benign pancreatic disease (BP-exo), and healthy people (N-exo) were isolated using an exosome isolation kit. The morphology and size of the exosomes were assessed using nanoparticle tracking analysis (NTA) and transmission electron microscopy (TEM) (Fig. 1A). In this study, patients with PC had higher protein concentrations than healthy individuals (Fig. 1B). Furthermore, the plasma exosomal markers CD63, CD9, and exosomal DNAJB11 were observed using western blotting (Fig. 1C). Moreover, the sizes of different groups showed no significant differences (Fig. 1D). The corresponding clinical information of the research group is provided in Additional file 1: Table S1. We collected plasma samples from patients with malignant pancreatic cancer (MP, *n* = 31), benign pancreatic disease (BP, *n* = 28; biliary pancreatitis [2]; acute pancreatitis [5]; chronic pancreatitis [4]; pancreatic cystadenoma [9]; and pancreatic islet cell tumor), and normal controls (N, *n* = 31). The samples were used to assess whether exosomal DNAJB11 could be a diagnostic biomarker for pancreatic cancer. The result showed that exosomal DNAJB11 was significantly higher in the MP group than the BP group (*P* < 0.01) and N (*P* < 0.0001) group (Fig. 1E). Furthermore, the receiver operating characteristic curve was obtained to assess the diagnostic role of exosomal DNAJB11 for pancreatic cancer. The areas under the curve (AUC) indicated the diagnostic ability between the MP and N groups (AUC 0.8044), the MP and BP groups (AUC 0.735), and the BP and N groups (AUC 0.7615) (Fig. 1F–H). Thus, exosomal DNAJB11 is a potential diagnostic biomarker of PC.

### Survival analysis and clinical features of DNAJB11 expression in PC tissue

**Fig. 2:**
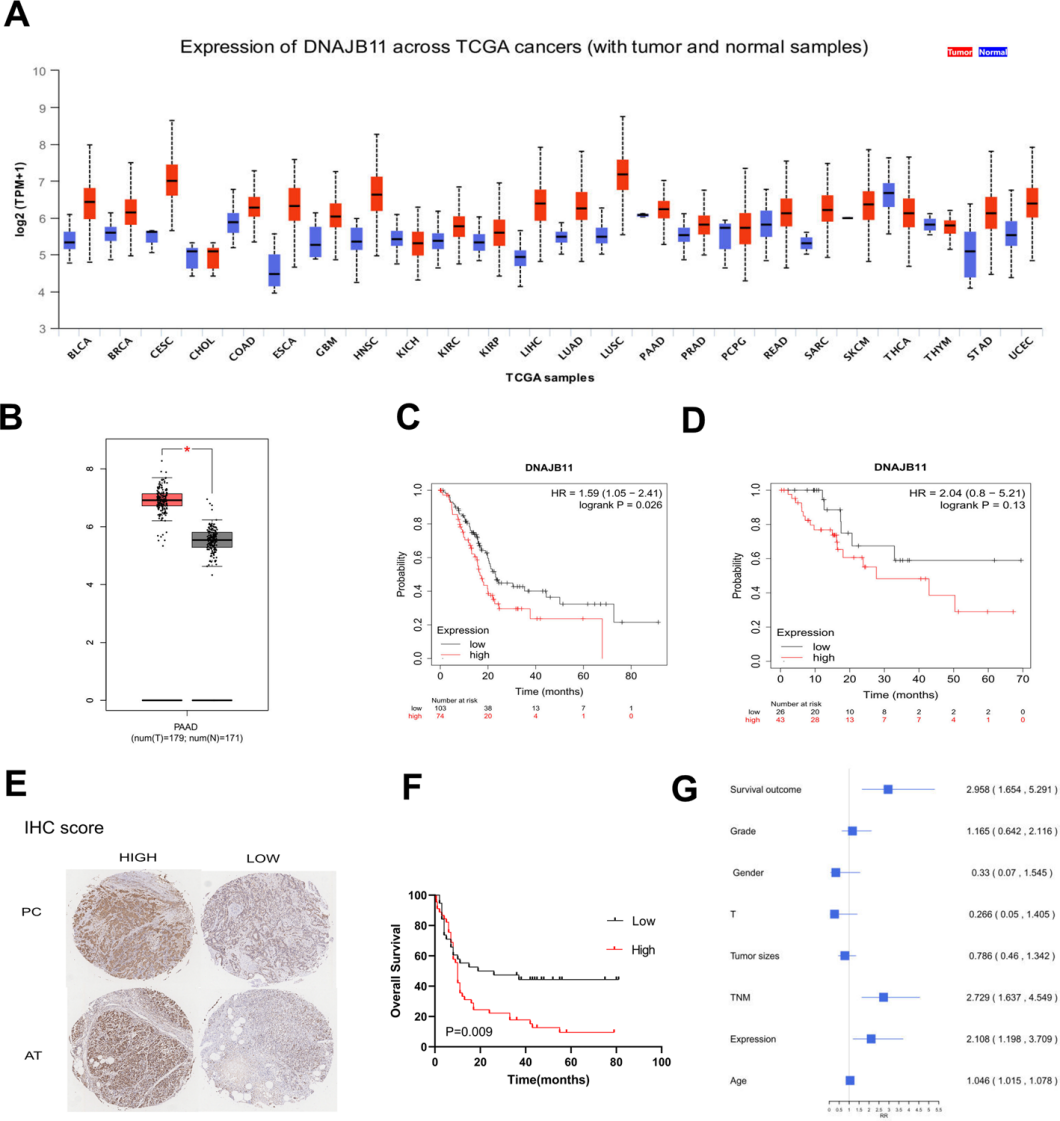
DNAJB11 expression is elevated in pancreatic cancer tissues and associated with a poor prognosis. **A** Expression levels of DNAJB11 in most cancers, as shown by the TCGA database. **B** DNAJB11 exhibits significantly higher expression in pancreatic cancer than in healthy individuals. **C** Higher DNAJB11 showed a poorer survival rate in patients with pancreatic cancer. **D** Alterations in DNAJB11 were less correlated with RFS in patients with pancreatic cancer. **E** Representative images of DNAJB11 staining in tumor tissues and adjacent tissues (AT). Scoring for the IHC is indicated. **F** Overall survival analysis of patients with pancreatic cancer with DNAJB11 expression by Kaplan–Meier analysis with log-rank test (**P < 0.01). **G** Multivariate cox proportional hazard analyses revealed that advanced TNM stage DNAJB11 expression contributed to poor OS

Multiple bioinformatics tools were performed for analyzing the clinical data on DNAJB11 from the GTEx database and TCGA. DNAJB11 was markedly expressed in most cancers, including PC by UALCAN and GEPIA (Fig. 2A, B). KM plotter analysis was used to analyze the relationship between DNAJB11 expression and survival; 74 patients with a high expression of DNAJB11 indicated poor overall survival (OS) compared with those with low expression (*P* = 0.026, Fig. 2C), but relapse-free survival (RFS) was not significantly different (*P* = 0.13) (Fig. 2D). To characterize DNAJB11 expression in PC, we purchased a PC tissue microarray containing 90 PC tissue samples and 56 matched adjacent paracancerous tissue samples. Integral images of immunohistochemical staining of the TMA with an anti-DNAJB11 were captured. Higher levels of DNAJB11 were observed in PC tissue, compared with the matched adjacent tissues. Corresponding staining of pancreatic cancer and adjacent tissues are shown in Fig. 2E. The immunohistochemical (IHC) score of every sample in the TMA was determined (Additional file 2: Table S2). DNAJB11 expression was significantly higher in PC compared with the adjacent tissues (Table 1). Subsequently, the effects of DNAJB11 expression on the pathological characteristics and prognosis of patients with PC were analyzed. The survival outcome of the patients was related to the DNAJB11 levels (Fig. 2F). Patients with high expression of DNAJB11 showed poorer OS than those with low expression of DNAJB11 (*P* < 0.009). To study the prognosis-related factors, we used multivariate Cox regression. The results showed that the expression of DNAJB11 was an independent risk factor for PC (Fig. 2G). The high DNAJB11 expression levels correlate with tumor size (Table 2). The results showed that DNAJB11 was overexpressed in PC and closely associated with the survival of patients.

**Table 1:** Differential expression of DNAJB11 in PDAC and adjacent tissues

	*n*	DNAJB11 expression		*P*-value
		Low	High	
Pancreatic cancer	56	23	33	0.037^*^
Adjacent tissues	56	34	22	

*Statistically significant (*P* < 0.05), ^#^fisher

**Table 2:** Correlation between DNAJB11 expression and clinicopathological characteristics

Variable	DNAJB11 expression		Total	*χ*^2^	*P*-value
	Low	High			
Age (years)				0.461	0.497
≤ 60	17	21	38		
> 60	24	22	46		
Sex				2.001	0.157
Female	12	19	31		
Male	29	24	53		
Grade				3.221	0.073
I/II	33	27	60		
III	8	16	24		
Tumor sizes (cm)				5.777	0.016
< 5	22	12	34		
≥ 5	19	31	50		
T stage				3.717	0.054
T1/T2	18	29	47		
T3/T4	22	15	37		
N stage					
N0	24	25	48	0.009	0.092
N1/N2	18	18	36		
M stage					
M0	40	43	83		0.488
M1	1	0	1		

### Exosomes containing DNAJB11 promote PC cell proliferation, migration, and invasion

**Fig. 3:**
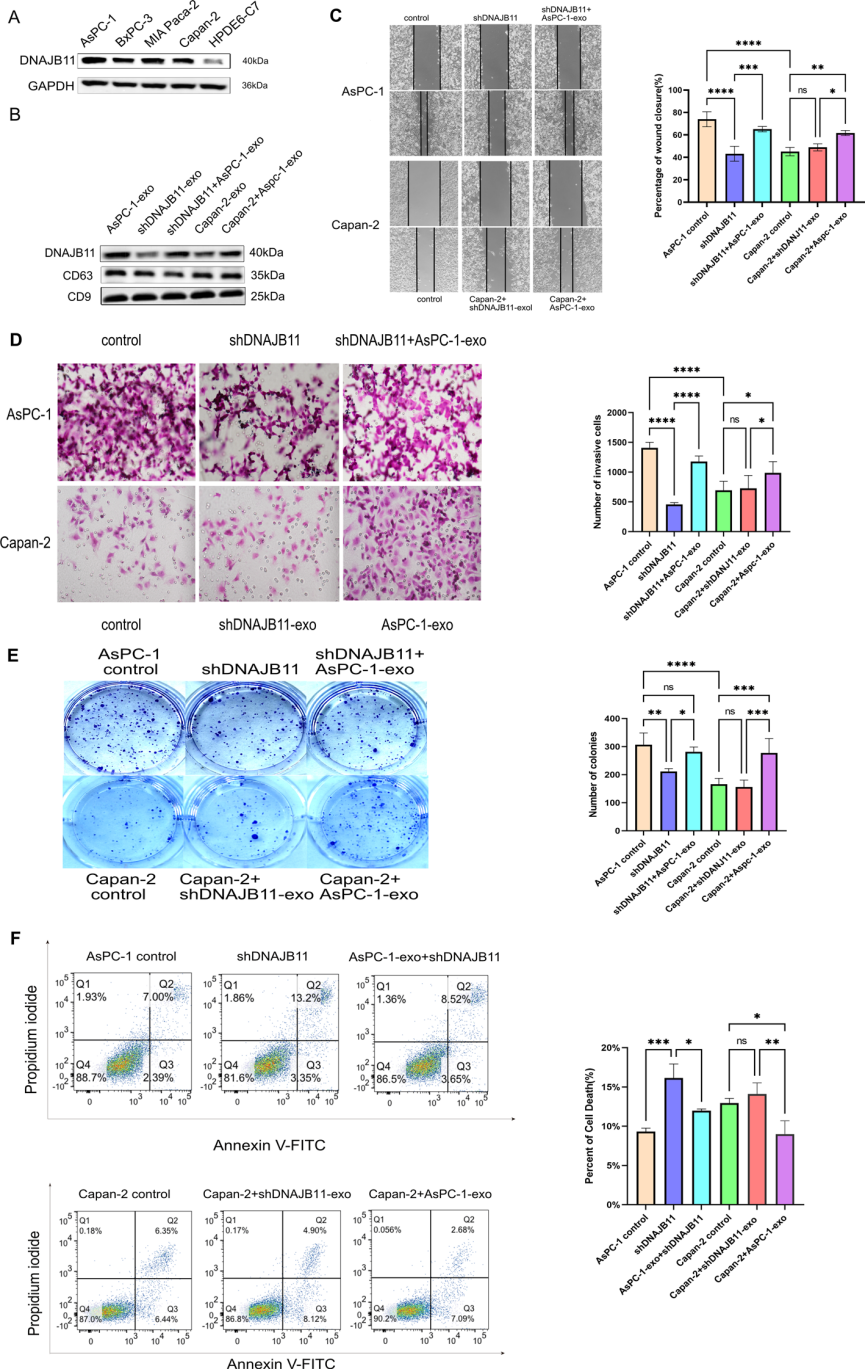
Exosomal DNAJB11 expression is associated with cell proliferation, invasion, and migration in pancreatic cancer cells. **A** Western blotting of DNAJB11 expression in pancreatic cancer cell lines and human normal pancreatic cell line. GAPDH was used as a control. Three independent experiments were performed. **B** PC cells were incubated with exosomes, and exosome markers CD63 and CD9 as well as exosomal DNAJB11 levels were measured using western blotting. Images of the wound healing assay (**C**), transwell (**D**), clone formation (**E**), and cellular apoptosis (**F**), and corresponding statistical analysis, respectively. After incubation with AsPC-1-exoor shDNAJB11-exo for 72 h, PC cell line migration, invasion, proliferation, and apoptosis were recorded (*P < 0.05, **P < 0.01, ***P < 0.001, ****P < 0.0001, one-way ANOVA). AsPC-1 and Capan-2 cell lines were the control group

To characterize the functional role of exosomal DNAJB11 during PC progression, first, DNAJB11 expression was measured in four human PC cell lines (AsPC-1, BxPC-3, MIA paca-2, and Capan-2) and a normal pancreatic cell line (HPDE6-C7) (Fig. 3A). Compared with the other cell lines, the expression of DNAJB11 was higher in the AsPC-1. Therefore, AsPC-1 cell lines were chosen for further assays, and the efficacy of DNAJB11 knockdown was determined by western blot analysis. We extracted the AsPC-1, shDNAJB AsPC-1, and Capan-2 cell exosomes, respectively. Furthermore, the shDNAJB AsPC-1 and Capan-2 cells were treated with AsPC-1-derived exosomes (50 µg/ml). The presence of exosomal markers and the corresponding expression of exosomal DNAJB11 in the different groups were determined by western blot analysis (Fig. 3B). The results indicated that DNAJB11 could be delivered into other cells through the exosomes. Further, we verified the effects of exosomal DNAJB11 in PC progression by comparing the functions of wild-type (WT) and DNAJB11-knockdown exosomes from AsPC-1 cells in vitro. The shDNAJB11 group showed poorer proliferation, migration, and invasion abilities than the control group. In addition, the abilities were recovered after treatment with AsPC-1-exo. Furthermore, exosomes containing DNAJB11 significantly stimulated proliferation, migration, and invasion compared with shDNAJB11-exo (Fig. 3C–E). shDNAJB11 group exhibited a significant increase in cellular apoptosis rate compared with AsPC-1. However, exosomal DNAJB11 significantly decreased the cellular apoptosis rates compared with Capan-2 (Fig. 3F).

### DNAJB11 mediates transcriptomic changes in PC cells

**Fig. 4:**
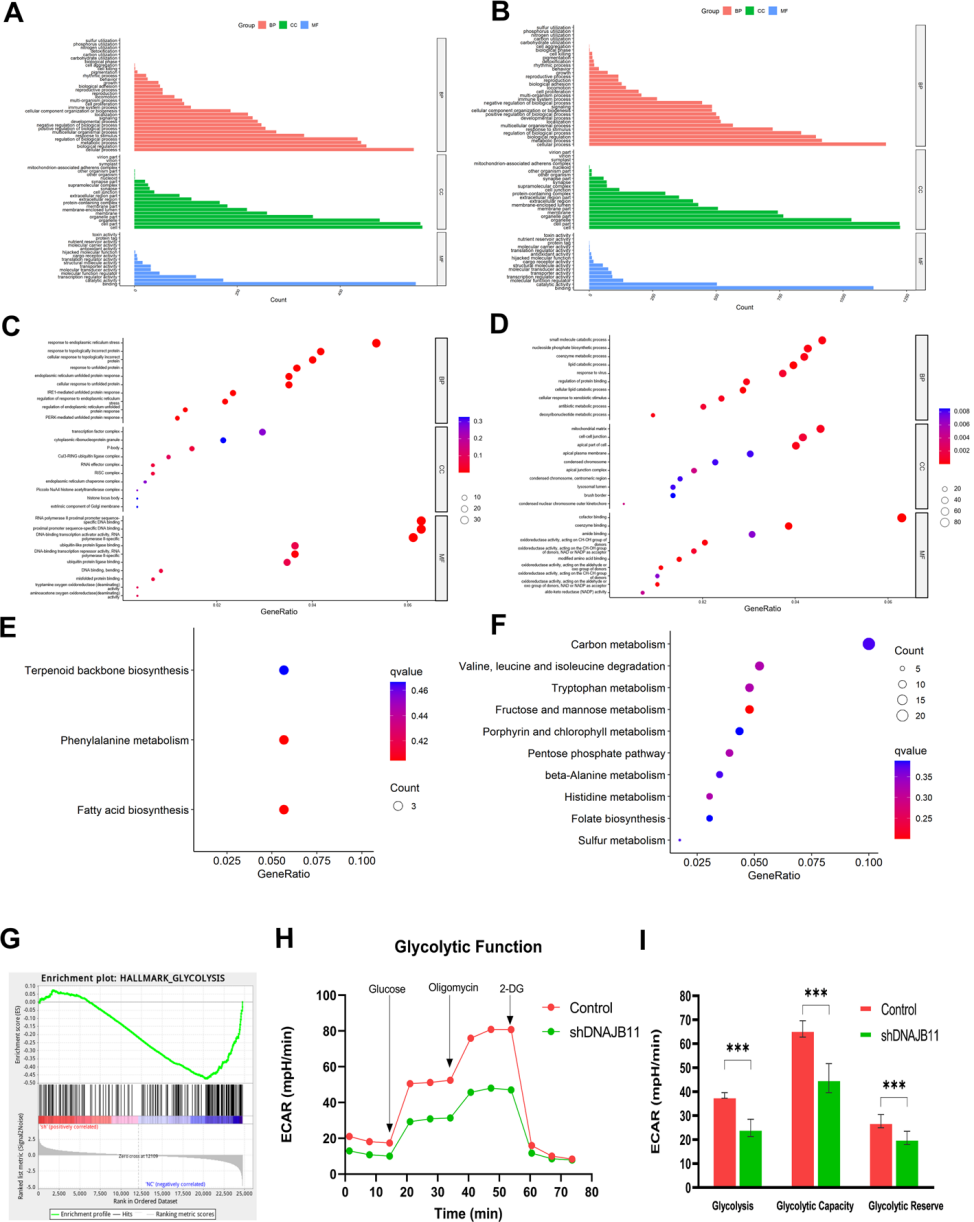
Bioinformatic analysis of WT and DNAJB11-knockdown transcriptomic sequencing identifies DNAJB11 as a regulator of glycolytic function. **A**–**D** Gene Ontology (GO) functional classification and subcellular functional annotation of DEGs (837 upregulated genes in WT and 1499 downregulated genes in DNAJB11-knockdown cell). **A** GO of upregulated genes. **C** GO analysis of top ten upregulated genes. **B** GO of downregulated genes. **D** GO analysis of top ten downregulated genes. **E** KEGG pathway analysis of top ten upregulated genes. **F** KEGG analysis of top ten downregulated genes. **G** Glycolysis was inhibited in the DNAJB11-knockdown groups using the analysis of Gene Set Enrichment Analysis (GSEA). **H**–**I** The glycolytic function was measured by Seahorse XF96 and indicated that DNAJB11 is involved in glycolysis. ***P < 0.001

To characterize DNAJB11-mediated transcriptomic changes in PC cells, transcriptomic sequencing (RNA-seq) was conducted after DNAJB11 knockdown. On analysis, a total of 24,760 expressed genes were detected, 837 genes were upregulated, and 1499 genes were downregulated (Additional file 3: Table S3). Gene Ontology enrichment analysis was performed on all the DEGs, and detailed information is provided in Additional file 2: Table S2. Volcano and clustering heat maps were performed on all the detected genes (Additional file 8: Fig S1B). ​ GO and KEGG analysis was used to analyze the biological processes (BP), cellular components (CC), molecular functions (MF), and enriched pathways (Fig. 4A–F). The top ten upregulated and downregulated enriched terms of DEGs in the BP, CC, and MF terms are shown in Fig. 4C, D.

Furthermore, 32 genes were enriched in ERS in the upregulated group, whereas 59 were enriched in the small-molecule catabolic process. In addition, KEGG analysis of DEGs enriched the pathway involved in cell metabolism (Fig. 4E, F). The results illustrate that DNAJB11 has an important role in cell metabolism. We performed the GSEA analyses based on the DEGs to further investigate the underlying molecular mechanisms. The DEGs were enriched for 50 hallmark gene sets (Additional file 4: Table S4), correlating with proliferation (HALLMARK_E2F_TARGETS) and interferon response (HALLMARK_ INTERFERON_GAMMA). The result indicated that the proliferation signature was linked with the gain of the inflammatory interferon response in the PC tissue [25]. Notably, the glycolysis gene set was ranked higher in the upregulated group (Fig. 4G). The Seahorse array was performed to analyze metabolism. Consistent with the GSEA results, DNAJB11 was involved in the energy metabolism process. Thus, inhibiting the DNAJB11 expression could reduce glycolysis (Fig. 4H, I, Additional file 5: Table S5). In addition, the TNFA-NFKB signaling pathway and RAS signaling pathway are presented in upregulated and downregulated groups. The observations suggested that DNAJB11 could regulate the progression of PC by increasing the RAS signaling pathway.

### CoIP-base proteomics analysis of DNAJB11

**Fig. 5:**
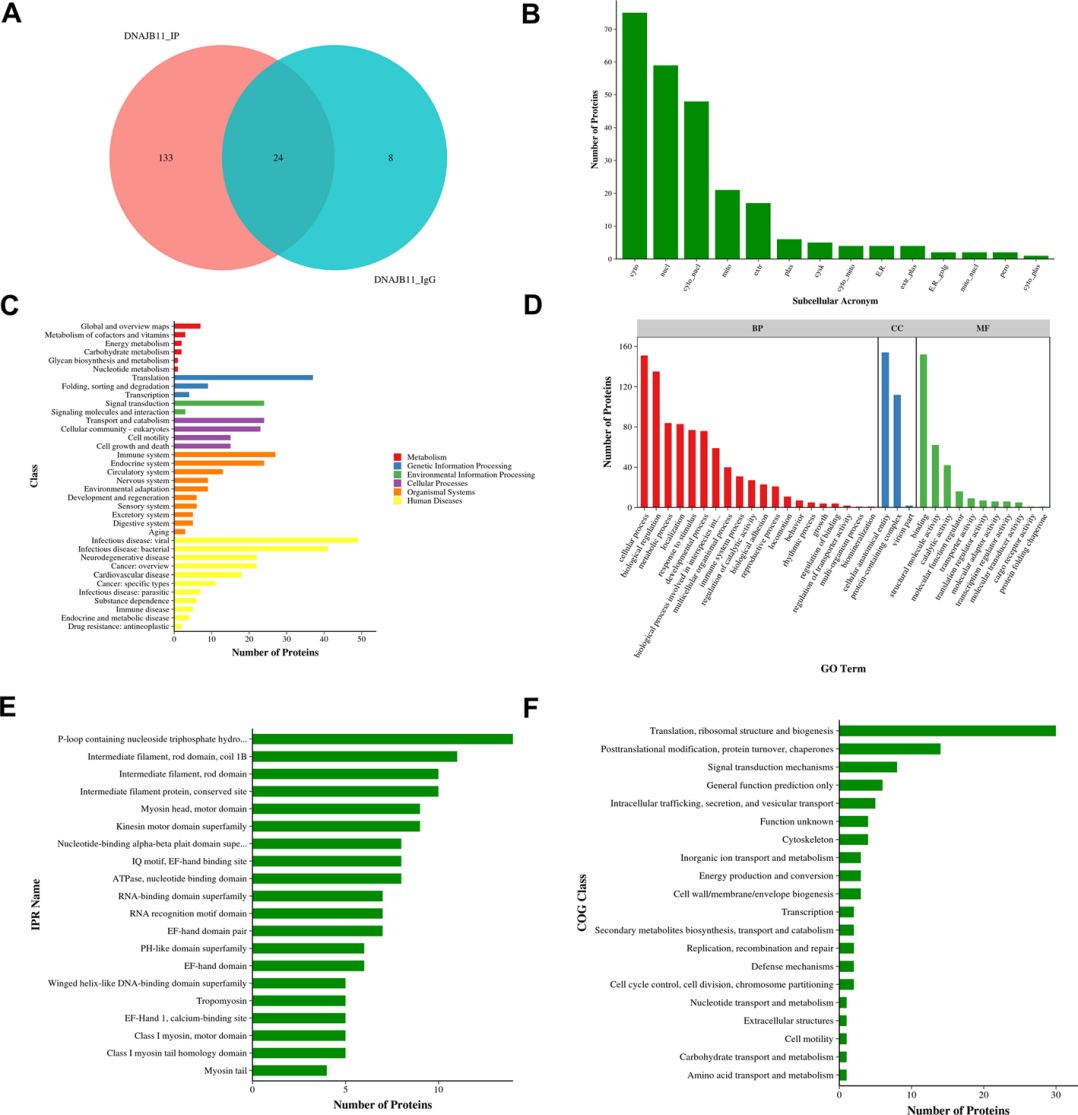
Function enrichment analysis of DNAJB11 co-expression proteins. **A** Venn diagram of proteins from IgG and DNAJB11 group. **B** The subcellular localization was predicted with the WoLF PSORT database. **C** KEGG analysis **D** GO analysis. **E** Protein domains were predicted with InterProScan. **F** Cluster of orthologous groups of proteins (COG) analysis was carried out for the identified proteins

To understand the DNAJB11 molecular mechanisms in PC cells, we performed a Co-IP-based proteomics assay to discover its interacting proteins. IgG served as a negative control for Co-IP. Two protein samples obtained from previous experiments were qualitatively analyzed by LC–MS/MS. On the basis of the search results, a Venn diagram was drawn (Fig. 5A). There were 157 proteins in the DNAJB11-IP group, and 32 in the IgG group (Table 3). Furthermore, 24 proteins were identified in both groups (Fig. 5A, Additional file 6: Table S6). In addition, 133 proteins in the DNAJB11-IP group were analyzed by five online bioinformatics databases (Fig. 5B–F, Additional file 7: Table S7). WoLF PSORT (https://wolfpsort.hgc.jp/) [26] was used to predict the localization of proteins (Fig. 5B). The results showed that DNAJB11-IP mostly exited in the cytosol and nucleus. The GO and KEGG analysis showed proteins enriched in translation, transport, and catabolism (Fig. 5C) as well as cellular process and binding, and structural molecule activity (Fig. 5D). Therefore, we ran InterProScan [27] to analyze domain architectures. Results showed that there were 14 proteins that include P-loop-containing nucleoside triphosphate hydrolase (Fig. 5E). The genes were placed into clusters of orthologous groups (COG) categories according to their functional annotation. Thirty-four proteins were enriched in the COG category for translation, ribosomal structure, and biogenesis (COG J), and 14 proteins were in the posttranslational modification, protein turnover, and chaperones (COG O) (Fig. 5F).

**Table 3:** Summary of protein identification information in Co-IP-based LC–MS/MS

Group	Total spectrum	Identified spectrum 2	Peptide	Protein	Unique protein
DNAJB11-IP	6482	1736	1044	157	119
DNAJB11-IgG	1465	312	189	32	23

### Blocking DNAJB11 induced ER stress and suppressed the EGFR signaling pathway

**Fig. 6:**
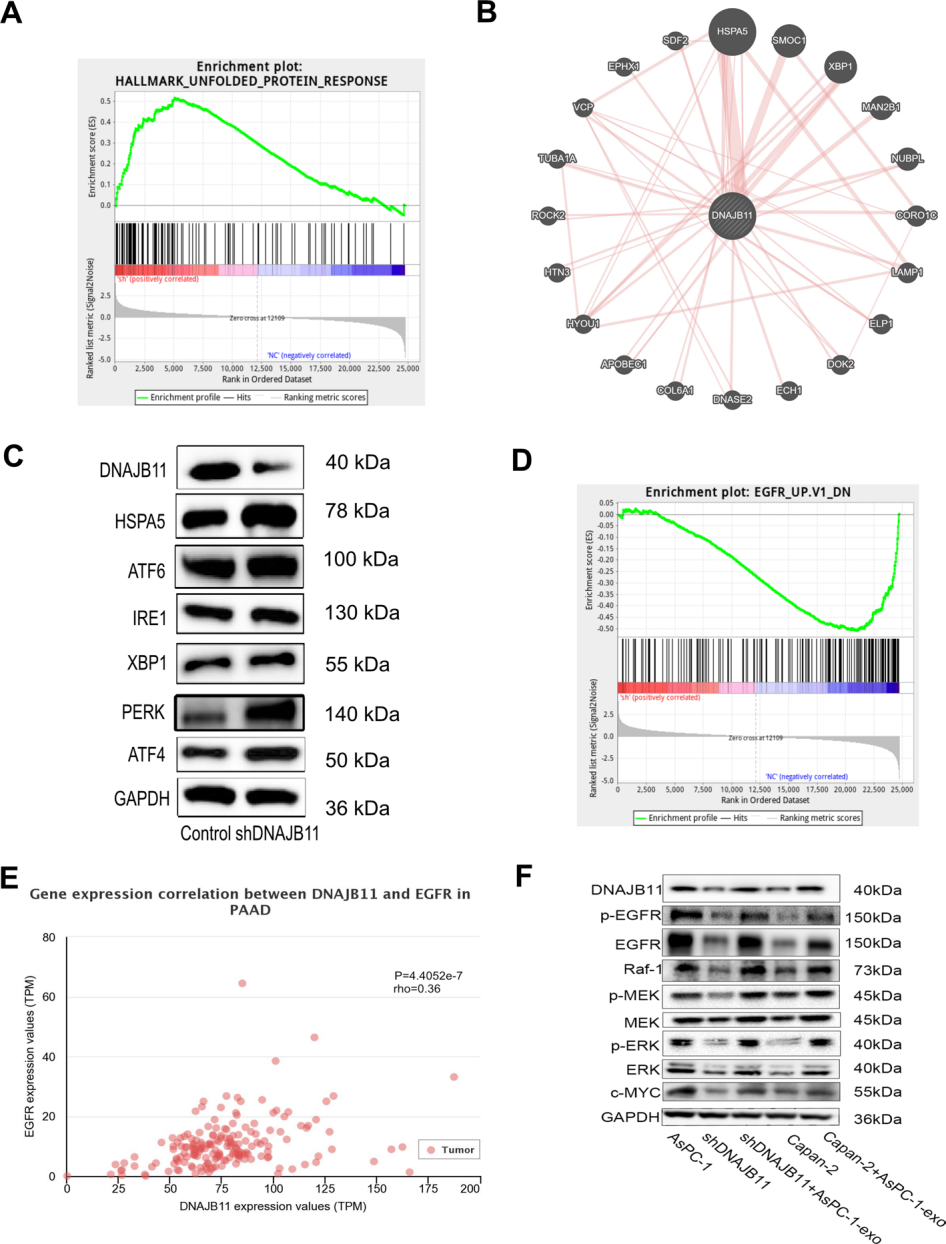
DNAJB11 inhibited ER stress activity by the PERK-ATF4 pathway and induced the EGFR/MAPK pathway. **A.** The UPR pathway was upregulated in the WT group by GSEA. **B** Functional network integration was performed by GeneMANIA. **C** The protein levels of DNAJB11, HSPA5, ATF6, IRE1, XBP1, PERK, ATF4, and GAPDH were measured by western blot analysis. **D** EGFR was inhibited in the DNAJB11-knockdown group by GSEA. **E** Correlation analysis of DNAJB11 and EGFR by UALCAN. **F** After incubation with exosomes, the protein levels of DNAJB11, EGFR, p-EGFR, Raf-1, MEK, p-MEK, ERK1/2, p-ERK1/2, c-MYC, and GAPDH were measured by western blot analysis

DNAJB11 plays an important role in the UPR. Our data showed that blocking DNAJB11 could induce ER stress by PERK-mediated UPR (Fig. 6A). The functional network integration was performed using GeneMANIA, and DNAJB11 was high compared with HSPA5 (Fig. 6B) [28]. The results were validated by western blot analysis (Fig. 6C). In the shDNAJB11 group, HSPA5, PERK, and ATF4 expression levels increased, but not ATF6, XBP1, and IRE1. Thus, blocking DNAJB11 activates ER stress through PERK-ATF4 of the UPR. Therefore, the mechanism by which exosomal DNAJB11 promotes cancer cell growth was verified. However, compared with AsPC-1 cells, we did not find a significant difference in the DNAJB11/PERK signaling pathway in shDNAJB11-AsPC-1 and Capan-2 cells after incubation with exosomal DNAJB11. This is probably because overexpression was more complex. Therefore, the UPR was regulated by another gene concurrently and not by DNAJB11 only. In addition, GSEA analyses revealed that DNAJB11 depletion could increase EGFR expression (Fig. 6D). Furthermore, our Co-IP-based MS data showed that DNAJB11 interacts with EGFR. UALCAN was also performed to analyze the correlation between DNAJB11 and EGFR (*P* < 0.001) (Fig. 6E). Combining the RNA-seq results, we hypothesized that DNAJB11 induced EGFR/RAF/MAPK signaling to promote cancer cell growth. Indeed, blocking DNAJB11 suppresses the RAF/MPAK signaling by reducing the EGFR expression level. Moreover, EGFR and p-EGFR protein levels were elevated in shDNAJB11-AsPC-1 and Capan-2 cells after the uptake of DNAJB11-exo (Fig. 6F). Significant differences were also seen in p-MEK and p-ERK protein expression in each experimental group compared with the corresponding control groups. Our data reveal that DNAJB11 induces the activation of the EGFR signaling pathway and suppresses the PERK-ATF4 ER stress signaling pathways.

### Exosomal DNAJB11 promotes pancreatic cancer growth in a nude mouse model

**Fig. 7:**
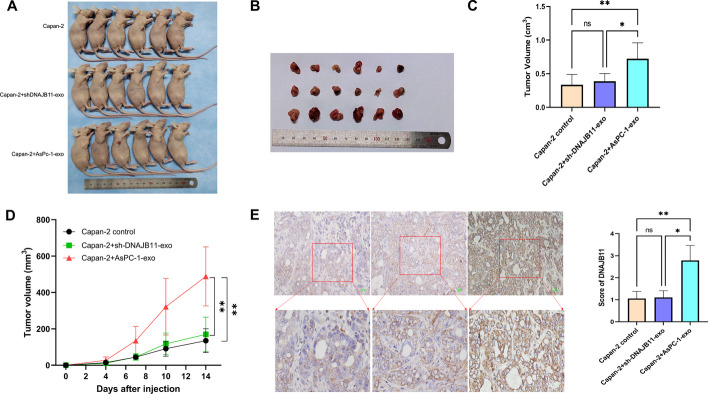
Exosomal DNAJB11 promotes pancreatic cancer growth in a xenograft model. **A** Subcutaneously implanted tumor 2 weeks after initial implantation. **B** Surgically excised tumor tissue. **C** Statistical analysis of the weight of the subcutaneously implanted tumor. **D** Growth curve of subcutaneously implanted tumors. **E** Representative image of immunofluorescence staining of DNAJB11 in a paraffin-embedded excised tumor tissue section. *P < 0.05, **P < 0.01; ***P < 0.001, one-way ANOVA

To further investigate the effect of exosomal DNAJB11 in vivo, subcutaneous tumors were generated in nude mice with 1 × 10^7^ Capan-2 cells. The nude mice treated with normal AsPC-1-exo showed rapid and larger tumor growth than those treated with shDNAJB11-exo (Fig. 7A–D). Furthermore, immunohistochemical assays showed that DNAJB11 expression was higher than in those injected with shDNAJB11-exo, indicating that exosomes enriched in DNAJB11 increased tumor growth (Fig. 7E).

## Discussion

Pancreatic cancer is an aggressive malignancy worldwide. Patients diagnosed with early-stage pancreatic cancer should receive therapy for a good clinical outcome [29, 30]. Early detection of PC is important in the prognosis and survival of patients with PC. In this study, we revealed that not only was the expression of DNAJB11 in the PC tissue high, but also exosomal DNAJB11 was higher in plasma exosomes from patients with PC than healthy individuals. Exosomal DNAJB11 and DNAJB11 may play an important role in PC development.

DNAJB11, a co-chaperone to HSPA5, is a soluble resident ER glycoprotein [31]. The N terminus of DNAJB11 was expressed in the cytosol and the nucleus, and the C terminus was targeted to the ER lumen and membrane-anchored [32, 33]. Thus, the conformation is vital in the process of unfolded or misfolded protein transfer. Besides, the N terminus includes the J domain, which provides a dynamic surface to form functional interactions with the HSPA5 [34]. Furthermore, the binding of DNAJB11 and HSPA5 could quickly protect the unfolded protein from aggregation, and DNAJB11 and HSPA5 are more frequently dispersed than other chaperones (e.g., ERdj4, ERdj5)[35]. Previous studies have reported that DNAJB11 is highly upregulated by unfolded secretory protein stress in the ER and could regulate a partial HSPA5’s gating activity to stimulate ATPase activity up to fivefold [36–38]. In addition, HSPA5 activates the UPR by increasing the probability that ERS sensors recognize unfolded proteins [39]. The UPR plays an important role in cancer progression [40, 41]. Previous studies established that DNAJB11 promotes cancer growth in hepatocellular carcinoma [42]. However, the interaction between DNAJB11 and UPR on PC has not been studied.

To elucidate further molecular mechanisms of DNAJB11 in patients with PC, IHC and TCGA database analysis of patients with PC were performed. DNAJB11 expression was positively correlated with advanced status and poor survival outcomes. Furthermore, DNAJB11 was associated with EGFR. As a result, DNAJB11 probably upregulated EGFR expression and directly promoted angiogenesis in PC tissues. In addition, there was a significant increase in DNAJB11 in the plasma exosomes from patients with PC. Hence, DNAJB11 overexpression contributed to the development and progression of PC.

In our study, we found that DNAJB11 reduced the expression of HSPA5 in pancreatic cancer cells. In shDNAJB11 cells, there was a corresponding increase in HSPA5 expression. However, in the Co-IP MS experiment, the direct interaction between DNAJB11 and HSPA5 was confirmed, as previously reported by other studies [33]. Furthermore, DNAJB11 expression enhanced ATP hydrolysis, thus inducing a conformational change in occupied HSPA5, which locks the unfolded protein, and DNAJB11 release from HSPA5 complexes well before folding is complete [37, 43]. DNAJB11 has “handcuff”-like function with respect to HSPA5. When DNAJB11 was overexpressed, occupied HSPA5 could not bind to other proteins. However, the binding of HSPA5 and DNAJB11 was more resistant to mutational disruption than previously identified for other chaperones. In shDNAJB11 cells, UPR activity was increased in our study. The depletion of DNAJB11 increased the possibility of HSPA5 interacting with the unfolded protein and inducing UPR to protect against apoptosis from cancer cells.

It is known that the ER is the quality controller of proteins, and long-term ERS produces large amounts of unfolded protein. The unfolded proteins can be refolded to generate functional proteins or, when they accumulate improperly folded proteins, can exceed the threshold and be degraded by proteasome or trigger cell death signals by activating UPR, a phenomenon also called imbalance of ER homeostasis [44]. Over decades, the potential role of DNAJB11 in human chronic diseases has been investigated [45, 46]. Cell survival under chronic ERS that develops hepatic carcinomas is a symptom of aggressive cancer [47]. Recently, it has been reported that DNAJB11 upregulation decreases the potential antitumor activity of celecoxib [48]. ER-associated degradation (ERAD) enables cancer cells to tolerate toxic glycoprotein stress, thereby helping them to survive [49]. Pancreatic cancer cells maintain homeostasis by inducing the repeated use of abnormally folded proteins in cells, contributing to the progression of tumor tissues. We demonstrated that DNAJB11 is involved in ERAD by increasing ATF4 expression. ATF4 promotes pancreatic cancer progression and the mechanism of gemcitabine resistance [50]. Thus, silencing DNAJB11 expression triggers cellular apoptosis.

In addition, we compared the metabolism of shDNAJB11 and that of the parent cell line. The knockdown group showed low glycolysis. In addition, hypoxia was another cellular response by ERS noted. In this study, a comprehensive analysis of our RNA-seq and Co-IP-based MS data, seven genes (*ALPP*, *CD55*, *CGN*, *GSN*, *ITGAV*, *TFRC*, and *VIL1*) were found in both groups. Transferrin receptor (TFRC) is important for iron uptake and is related to ferroptosis, a non-apoptotic form of cell death. The complex relationship between DNAJB11 and TFRC in pancreatic cancer needs to be elucidated in future studies.

Therefore, we provided new insight into DNAJB11, identifying it as an exosome with important extracellular biological function. DNAJB11 in exosomes was protected from degradation. Like a sponge, it took up extracellular unfolded proteins and performed its function. However, the mechanisms of exosome–recipient cell interaction are unclear. In this study, exosomal protein altered the ability of invasion and metastasis in receipt cells. Previous studies report a similar molecular mechanism, that DNAJB11 is directly co-secreted with unfolded proteins to the extracellular environment and maintains proteostasis [51, 52]. Exosomal DNAJB11 activated the EGFR/MAPK signaling pathway in targeted PC cells. DNAJB11 is expressed in the upregulated differential expression group both intracellularly and extracellularly. Therefore, it is conceivable that interference with DNAJB11 can improve the tumor cells and their surrounding microenvironment simultaneously. This study also has certain limitations. For example, there is no description of how exosomes are transported into another cell. The different types of exosome were used to treat corresponding cells, and differences in function were assessed. The differences in function observed may be due to differential expression of DNAJB11. Hence, exosomal DNAJB11 is a potential biomarker for PC development.

## Conclusion

In summary, exosomal DNAJB11 is crucial in maintaining extracellular signaling, altered metabolic behavior, and adhesion properties of pancreatic cancer cells. Exosomal DNAJB11 may be a potential biomarker in PC development and progression. However, DNAJB11 is a crucial regulator upon ER stress and negatively controls the UPR signaling pathway. DNAJB11 promoted cancer development through the EGFR/MAPK signaling pathway, providing new insight into the development of pancreatic cancer. DNAJB11 inhibitors have been proposed as targets to impair the survival of cancer cells.

## Supplementary Information

**Additional file 1:** **Table S1.** Clinical information of the 90 patients run through the ELISA assay.

**Additional file 2: Table S2.** Clinical and pathologic information of patients in TMA.

**Additional file 3: Table S3.** A complete list of all genes identified in the transcriptome sequencing.

**Additional file 4: Table S4.** Enrichment analysis of DEGs.

**Additional file 5:** **Table S5.** The results of the Seahorse assay.

**Additional file 6:** **Table S6.** List of proteins identified in CoIP-based LC–MS/MS.

**Additional file 7:** **Table S7.** Enrichment analysis of proteins identified in CoIP-seq.

**Additional file 8:** **Figure S1.** The flow chart of transcriptome sequencing.

## Data Availability

All data generated or analyzed during this study are included in this published article and its Additional files.

## Abbreviations

PC: Pancreatic cancer
UPR: Unfolded protein response
ER: Endoplasmic reticulum
TME: Tumor microenvironment
DNAJB11: DnaJ Heat Shock Protein Family (Hsp40) Member B11
HSPA5: Heat Shock Protein Family A (Hsp70) Member 5
GSEA: Gene Set Enrichment Analysis
TCGA: The Cancer Genome Atlas
ERAD: Endoplasmic-reticulum-associated degradation
TEM: Transmission electron microscopy
TMA: Tissue microarray
DEGs: Differentially expressed genes
LC–MS/MS: Liquid chromatography with tandem mass spectrometry
CoIP: Co-immunoprecipitation
ECAR: Extracellular acidification rates
GEPIA: Gene expression profiling interactive analysis
GTEx: Genotype-Tissue Expression
NTA: Nanoparticle tracking analysis
