# Supplementary material for: Exosomal DNAJB11 promotes the development of pancreatic cancer by modulating the EGFR/MAPK pathway
Source: Cell Mol Biol Lett. 2022 Oct 8;27:87. doi: 10.1186/s11658-022-00390-0 (PMC9548179; doi:10.1186/s11658-022-00390-0)
Supplement: Supplementary file 1 — Additional file 1: Table S1. Clinical information of the 90 patients run through the ELISA assay. [file 11658_2022_390_MOESM1_ESM.pdf]

Exosomal DNAJB11 promotes the development of pancreatic cancer by activating EGFR/MAPK signaling pathway and regulating Endoplasmic Reticulum Stress

Peng Liu[1], Lingming Kong[1], Fuqiang Zu1, Hui Chen1,Xiaoli Yin[2,*], Xiaodong Tan[1,*]

1 Department of general surgery, Shengjing Hospital, China Medical University, Shenyang 110004, China
2 Department of Radiology, Shengjing Hospital of China Medical University, Shenyang 110004, China
*Corresponding Author: Xiaodong Tan, 1st Department of general surgery, Shengjing Hospital, China Medical University, Shenyang 110004, China. E-mail:tanxdcmu@163.com.
Xiaoli Yin: Department of Radiology, Shengjing Hospital of China Medical University, Shenyang 110004, China, E-mail: yinxl057728@163.com.
These authors have contributed equally to this work.

Title: the information of Purified exosomes

Patient number	Admission diagnosis	Admission number	Age	Gender	TNM staging	Protein level by WB（ng/ul）
1	Pancreatic cancer	2210150	45	Female	T3N1M0	6446
2	Pancreatic cancer	2218518	73	Male	T3N0M0	4563
3	Pancreatic cancer	2220123	51	Female	T1N1M0	5313
4	Pancreatic cancer	2220529	56	Female	T3N1M0	4155
5	Pancreatic cancer	2284331	45	Male	T3N0M0	4705
6	Pancreatic cancer	1282143	50	Male	T2N1M0	4321
1	Benign pancreatic disease	2214144	42	Female		5980
2	Benign pancreatic disease	2213994	30	Male		4813
3	Benign pancreatic disease	2215704	78	Female		5080
4	Benign pancreatic disease	2217481	63	Male		5480
5	Benign pancreatic disease	2167617	52	Male		4989
6	Benign pancreatic disease	2268805	36	Male		5243
1	Healthy people		39	Female		1746
2	Healthy people		49	Female		2771
3	Healthy people		63	Male		3013
4	Healthy people		65	Female		2631
5	Healthy people		44	Male		2738
6	Healthy people		38	Female		2462

Exosomal DNAJB11 promotes the development of pancreatic cancer by activating EGFR/MAPK signaling pathway and regulating Endoplasmic Reticulum Stress

Peng Liu[1], Lingming Kong[1], Fuqiang Zu1, Hui Chen1,Xiaoli Yin[2,*], Xiaodong Tan[1,*]

1 Department of general surgery, Shengjing Hospital, China Medical University, Shenyang 110004, China
2 Department of Radiology, Shengjing Hospital of China Medical University, Shenyang 110004, China
*Corresponding Author: Xiaodong Tan, 1st Department of general surgery, Shengjing Hospital, China Medical University, Shenyang 110004, China. E-mail:tanxdcmu@163.com.
Xiaoli Yin: Department of Radiology, Shengjing Hospital of China Medical University, Shenyang 110004, China, E-mail: yinxl057728@163.com.
These authors have contributed equally to this work.

Title: the information of malignant pancretic cancer

Patient number	Admission number	Age	Gender	Admission diagnosis	TNM staging	CA19-9（U/ml）	450 OD value
1	2210150	45	Female	Pancreatic cancer	T3N1M0	631.9	1.520
2	2218518	73	Male	Pancreatic cancer	T3N0M0	>1000	0.695
3	2220123	51	Female	Pancreatic cancer	T1N1M0	22.73	1.507
4	2220529	56	Female	Pancreatic cancer	T3N1M0	39.98	1.498
5	2220833	60	Female	Pancreatic cancer	T3N2M0	>1000	1.590
6	2224117	53	Female	Pancreatic cancer	T2N0M0	73.33	1.728
7	2223982	59	Male	Pancreatic cancer	T3N1M0	51.82	1.224
8	1282143	50	Male	Pancreatic cancer	T2N1M0	899.1	1.308
9	2256684	51	Male	Pancreatic cancer	T3N0M0	131.7	1.250
10	2245214	65	Male	Pancreatic cancer	T2N0M0	200.1	0.184
11	2260900	67	Male	Pancreatic cancer	T2N0M0	241.9	1.681
12	2258438	57	Male	Pancreatic cancer	T4N0M0	185.5	1.179
13	2254405	68	Male	Pancreatic cancer	T2N0M0	5.02	0.843
14	2246083	52	Female	Ampullar cancer	T2N2M0	43.76	0.860
15	2275845	62	Female	Pancreatic cancer	T3N0M0	0.622	1.229
16	2280528	72	Female	Pancreatic cancer	T3N2M0	420.5	1.396
17	2283722	52	Male	Pancreatic cancer	T2N0M0	59.92	0.174
18	2280528	73	Female	Pancreatic cancer	T2N1M0	420.5	0.903
19	2284051	59	Male	Pancreatic cancer	T2N0M0	822.6	1.416
20	2284331	45	Male	Pancreatic cancer	T3N0M0	86.41	1.223
21	2284479	60	Female	Pancreatic cancer	T2N0M0	52.57	0.745
22	2235934	46	Male	Pancreatic cancer	T4N0M0	5.81	1.349
23	2245214	65	Male	Pancreatic cancer	T2N0M0	200.1	1.816
24	2285687	63	Female	Pancreatic cancer	T2N0M0	88.88	0.847
25	2289258	61	Female	Pancreatic cancer	T2N0M0	118.1	0.950
26	2291064	57	Female	Pancreatic cancer	T2N0M0	0.966	0.594
27	2291087	63	Female	Pancreatic cancer	T2N0M0	12.77	0.978
28	2335529	78	Female	Pancreatic cancer	T2N0M0	28.09	2.342
29	2945842	66	Female	Pancreatic cancer	T2N0M0	274.7	1.059
30	2337173	58	Male	Pancreatic cancer	T2N0M1	476.2	1.134
31	2337607	49	Male	Pancreatic cancer	T4N1M1	361.7	1.667

Exosomal DNAJB11 promotes the development of pancreatic cancer by activating EGFR/MAPK signaling pathway and regulating Endoplasmic Reticulum Stress

Peng Liu[1], Lingming Kong[1], Fuqiang Zu1, Hui Chen1,Xiaoli Yin[2,*], Xiaodong Tan[1,*]

1 Department of general surgery, Shengjing Hospital, China Medical University, Shenyang 110004, China
2 Department of Radiology, Shengjing Hospital of China Medical University, Shenyang 110004, China
*Corresponding Author: Xiaodong Tan, 1st Department of general surgery, Shengjing Hospital, China Medical University, Shenyang 110004, China. E-mail:tanxdcmu@163.com.
Xiaoli Yin: Department of Radiology, Shengjing Hospital of China Medical University, Shenyang 110004, China, E-mail: yinxl057728@163.com.
These authors have contributed equally to this work.

Title: the information of benign pancreatic disease

Patient number	Admission number	Age	Gender	Admission diagnosis	CA19-9（U/ml）	450 OD value
1	2223931	69	Male	Pancreatic cystadenoma	8.7	0.6623
2	2224073	65	Female	Pancreatic cystadenoma	56.4	0.6673
3	2243316	39	Female	Pancreatic mass	9.2	0.6093
4	2259781	34	Female	Pancreatic mass	3.3	1.6853
5	2263279	58	Male	Pancreatic mass	10.9	0.6183
6	2263806	34	Female	Pancreatic cystadenoma	11.2	0.7923
7	2261264	58	Male	Pancreatic cystadenoma	17.6	1.1833
8	2265117	32	Female	Pancreatic cystadenoma	7.7	0.7653
9	2268278	69	Female	Pancreatic mass	9.4	0.6363
10	2268772	65	Female	Pancreatic mass	12	1.7683
11	2277987	34	Female	Pancreatic mass	9.7	0.9233
12	2271477	45	Female	Pancreatic mass	7.9	0.8843
13	2269807	67	Male	Pancreatic cystadenoma	14.9	0.7943
14	2280627	52	Female	Pancreatic cystadenoma	6.3	0.9483
15	2285948	49	Female	Pancreatic cystadenoma	16.27	0.7293
16	2010616	58	Female	Pancreatic mass	46.09	0.6143
17	2022268	66	Female	Pancreatic cystadenoma	6.31	0.6643
18	2214144	42	Female	Chronic pancreatitis	7.2	0.6923
19	2213994	30	Male	Chronic pancreatitis	4.3	0.8043
20	2215704	78	Female	Acute pancreatitis	206.5	1.2633
21	2217481	63	Male	Acute pancreatitis	18.2	0.9663
22	2167617	52	Male	Chronic pancreatitis	14.8	0.5873
23	2268805	36	Male	Biliary pancreatitis	12	1.7303
24	2280685	56	Male	Acute pancreatitis	157.3	0.9823
25	2227408	61	Male	Acute pancreatitis	11.5	0.7893
26	2284726	62	Male	Acute pancreatitis	6.9	0.7843
27	1916883	61	Male	Chronic pancreatitis	70.11	0.5603
28	2015888	48	Male	Biliary pancreatitis	22.26	0.6133

Exosomal DNAJB11 promotes the development of pancreatic cancer by activating EGFR/MAPK signaling pathway and regulating Endoplasmic Reticulum Stress
Peng Liu[1], Lingming Kong[1], Fuqiang Zu1, Hui Chen1,Xiaoli Yin[2,*], Xiaodong Tan[1,*]

1 Department of general surgery, Shengjing Hospital, China Medical University, Shenyang 110004, China
2 Department of Radiology, Shengjing Hospital of China Medical University, Shenyang 110004, China
*Corresponding Author: Xiaodong Tan, 1st Department of general surgery, Shengjing Hospital, China Medical University, Shenyang 110004, China. E-mail:tanxdcmu@163.com.
Xiaoli Yin: Department of Radiology, Shengjing Hospital of China Medical University, Shenyang 110004, China, E-mail: yinxl057728@163.com.
These authors have contributed equally to this work.

Title: the information of healthy control

Number	Gender	Age	450 OD value
1	Female	22	0.438
2	Female	39	0.518
3	Female	49	0.353
4	Male	63	0.372
5	Female	65	0.598
6	Male	44	0.642
7	Female	38	0.643
8	Female	51	0.302
9	Female	64	0.382
10	Male	26	0.425
11	Female	23	1.401
12	Male	22	0.238
13	Male	28	0.419
14	Male	30	1.395
15	Male	24	0.349
16	Male	66	1.529
17	Female	51	0.384
18	Female	54	0.330
19	Female	54	0.238
20	Female	33	0.305
21	Female	59	0.389
22	Male	31	0.192
23	Female	37	0.343
24	Female	36	0.329
25	Male	58	1.337
26	Male	63	1.362
27	Male	76	0.550
28	Male	51	0.502
29	Male	60	1.186
30	Female	37	0.902
31	Female	32	0.985
