# Supplementary figures and images for: Exosomal DNAJB11 promotes the development of pancreatic cancer by modulating the EGFR/MAPK pathway
Source: Cell Mol Biol Lett. 2022 Oct 8;27:87. doi: 10.1186/s11658-022-00390-0 (PMC9548179; doi:10.1186/s11658-022-00390-0)

**A**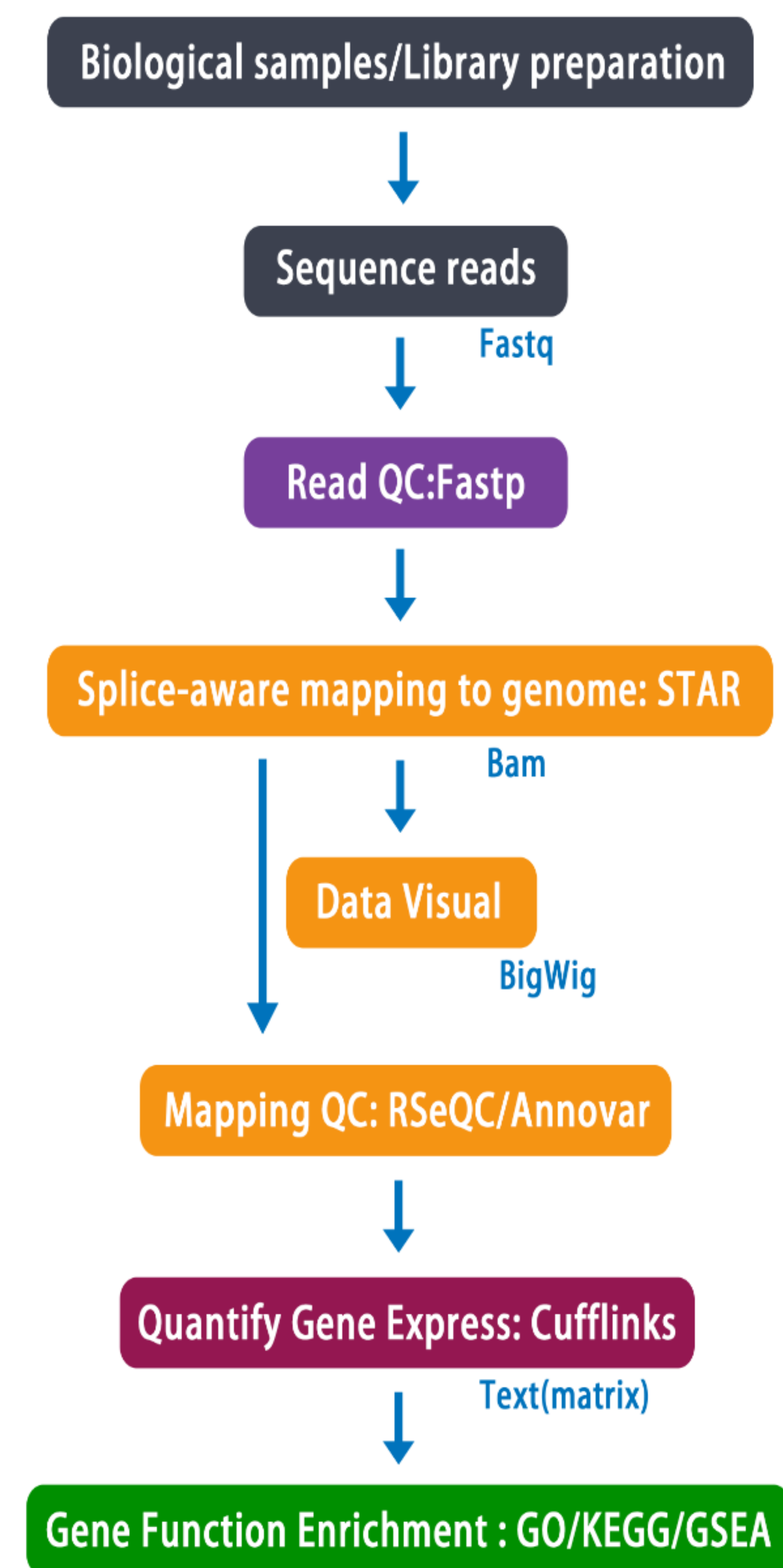**B**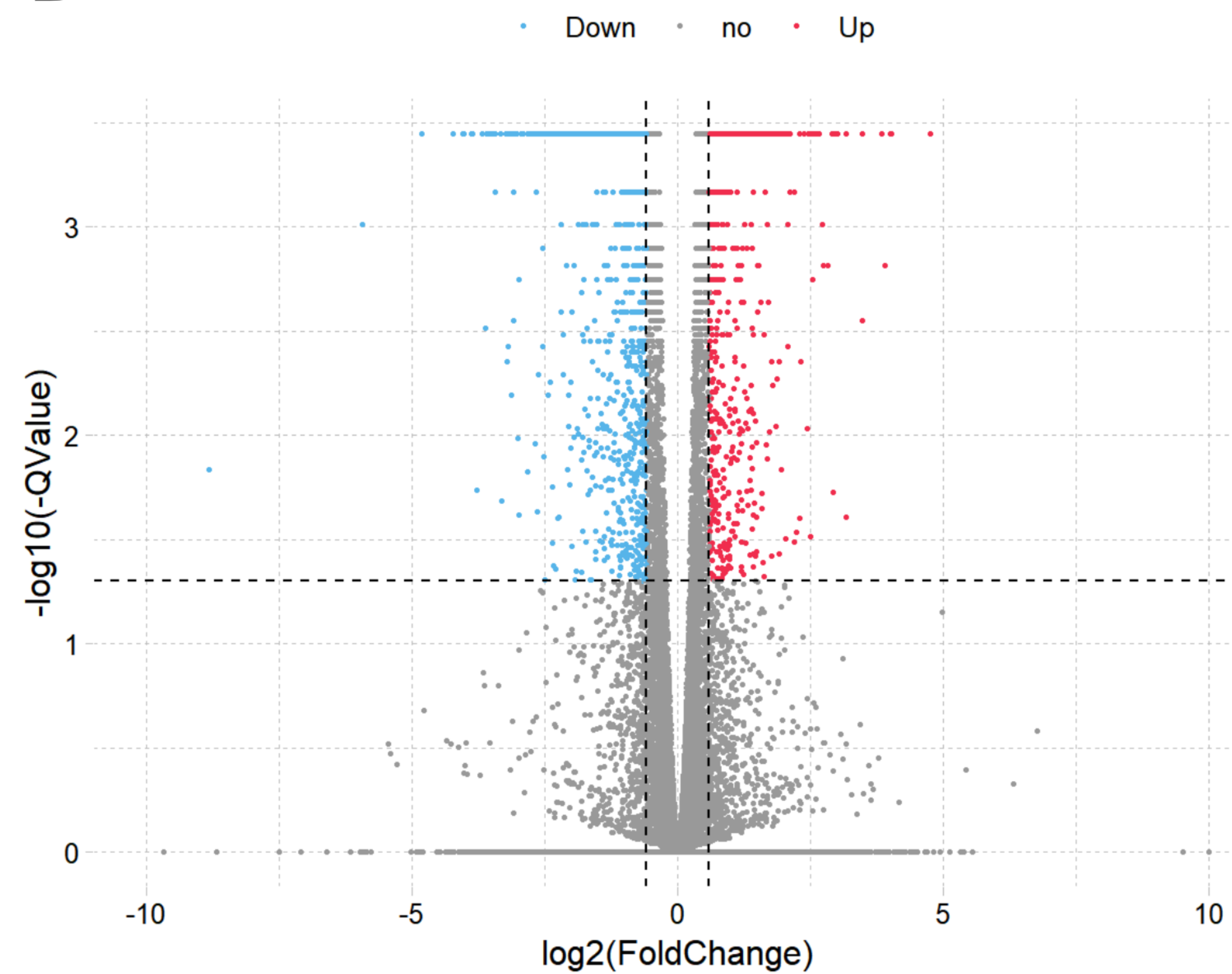**C**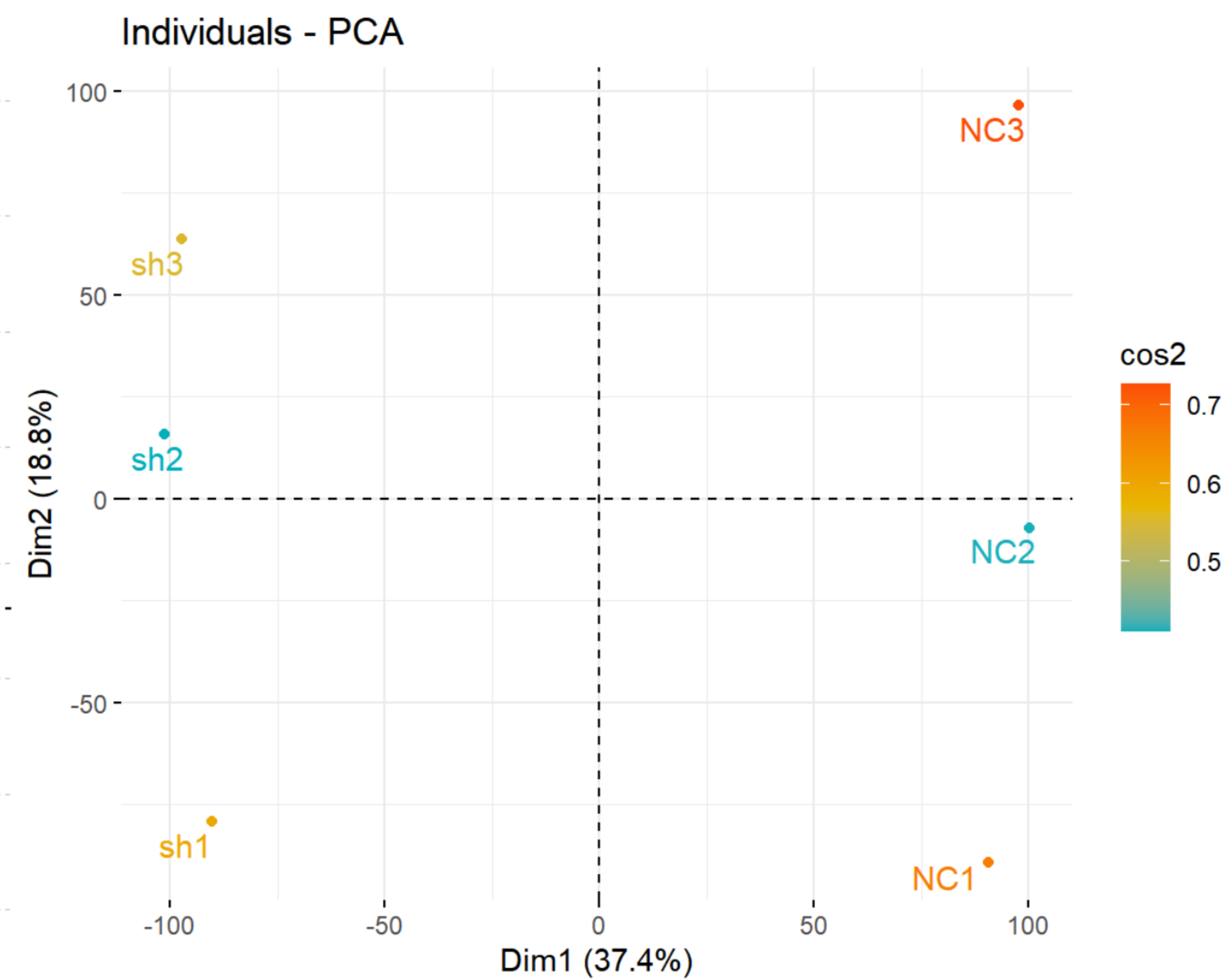

Supplement: Supplementary file 8 — Additional file 8: Figure S1. The flow chart of transcriptome sequencing. [file 11658_2022_390_MOESM8_ESM.pdf]
